# Hypoperfusion of the Left Insula, Operculum, and Putamen on Technetium-99m Ethyl Cysteinate Dimer (99mTc-ECD) Single-Photon Emission Computed Tomography in Patients With Mild Cognitive Impairment and Early Alzheimer's Disease

**DOI:** 10.7759/cureus.73184

**Published:** 2024-11-06

**Authors:** Hiroki Honda, Yasuhiro Watanabe, Takenobu Murakami, Mika Uemoto, Shinichiro Kitao, Shinya Fujii, Kiyotaka Nemoto, Ritsuko Hanajima

**Affiliations:** 1 Department of Neurology, Tottori University, Yonago, JPN; 2 Faculty of Medicine, Division of Radiology, Department of Multidisciplinary Internal Medicine, Tottori University, Yonago, JPN; 3 Faculty of Medicine, Division of Radiology, Department of Multidisciplinary Internal Medicine, Tottori University, Tottori, JPN; 4 Department of Psychiatry, University of Tsukuba, Tsukuba, JPN

**Keywords:** alzheimer's disease (ad), hypoperfusion, mild cognitive impairment (mci), spect, statistical parametric mapping

## Abstract

Objective: The easy Z-score imaging system (eZIS) objectively interprets brain perfusion. Using eZIS-processed images, we observed decreased regional cerebral blood flow (rCBF) in the left putamen of several patients with forgetfulness. This study aimed to examine this decrease using statistical image analysis.

Methods: Cerebral perfusion single-photon emission computed tomography (SPECT) was performed on patients with mild cognitive impairment (MCI) and early Alzheimer’s disease (AD). Normalized and corrected SPECT images were compared between patients and controls using Statistical Parametric Mapping software (SPM12, The Wellcome Center for Human Neuroimaging, UCL Queen Square Institute of Neurology, London, UK). Eigenvariate values of clusters with significantly low and high rCBF were obtained. Principal component analysis (PCA) was then used to examine the relationships among these clusters.

Results: We observed decreased rCBF in the left insula, operculum, and putamen, indicating that the reduction extended laterally from the initial eZIS-based findings. We obtained eight decreased and seven increased eigenvariate values for abnormal rCBF clusters. PCA revealed that the left insula, operculum, and putamen were the most influential principal components, along with the posterior cingulate and precuneus cortices. The eigenvariate values in these regions did not correlate with sex, diagnosis (AD or MCI), or cognitive scores.

Conclusions: We reported the decreased rCBF in the left insula, operculum, and putamen. However, its clinical significance beyond the associations with forgetfulness and its natural radiological course remains unclear.

## Introduction

Single-photon emission computed tomography (SPECT) provides valuable information for diagnosing neurodegenerative dementias. The posterior cingulate cortex, precuneus, and parietal lobe show reduced regional cerebral blood flow (rCBF) in Alzheimer’s disease (AD) [1,2], whereas occipital hypoperfusion with relative sparing of the posterior cingulate cortex, known as the “cingulate island sign” is a characteristic pattern in dementia with Lewy bodies (DLB) [3]. The easy Z-score imaging system (eZIS) is a software that visualizes rCBF reductions by comparing an individual patient’s rCBF image data with a database of normal rCBF images, projecting calculated Z-scores onto magnetic resonance images (MRI) of a standardized brain [4]. Using a standard brain phantom, eZIS facilitates the sharing of databases and imaging data between institutions, even if different scanning devices are used.

Among the patients who visited our hospital with forgetfulness as their main complaint, we observed decreased rCBF in the left putamen using the eZIS analysis. The decreases statistically indicated a reduction in rCBF of more than two standard deviations. The incidence rate of this finding was notably high to ignore; hence, we retrospectively and methodologically analyzed the rCBF in the corresponding regions. This study presents and discusses these findings.

## Materials and methods

Subjects

**Figure 1:**
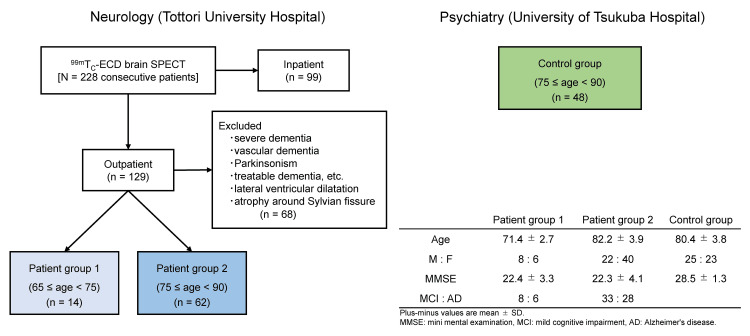
Schematic presentation of subjects and groups. Outpatients were screened and diagnosed with mild cognitive impairment (MCI) or early Alzheimer’s disease (AD) at Tottori University Hospital. The patients were classified into two groups: patient group 1 (light blue), comprising individuals aged 65 to less than 75 years; and patient group 2 (blue), comprising individuals aged 75 to less than 90 years. Control data were provided by the psychiatry department at the University of Tsukuba Hospital. The control group (green) consisted of individuals aged 75 to 89 years, matched for age with patient group 2. SPECT: single-photon emission computed tomography, 99mTc-ECD: technetium-99m ethyl cysteinate dimer.

This study included 76 patients aged 65-89 years who visited our outpatient clinic at the Department of Neurology, Tottori University Hospital, with forgetfulness as their chief complaint. All patients underwent technetium-99m ethyl cysteinate dimer (99mTc-ECD) SPECT imaging between January 2019 and March 2020. This retrospective observational study was approved by the Ethics Committee of Tottori University Hospital (18A040). The patient selection and final cohort used in the study are summarized in Figure 1.

The included patients had mild cognitive impairment (MCI) or early AD, clinically diagnosed according to respective criteria: the revised criteria for MCI [5] and for AD [6]. The patients were categorized into two groups according to their age, as follows: patient group 1 (n = 14, mean age at examination: 71.4 ± 2.7 years) and patient group 2 (n = 62, mean age: 82.2 ± 3.9 years).

The exclusion criteria included severe dementia, vascular dementia, Parkinsonism, other diseases causing dementia, and other neurological conditions (e.g., migraines). Patients with MRI-confirmed cerebrovascular lesions, dilatated Virchow-Robin spaces, dilatated lateral ventricles, and cortical or subcortical atrophy around the Sylvian fissure were also excluded (Figure 1). Special attention was given to structures around the Sylvian fissure and lateral ventricles, which were closely examined by neuroradiologists to avoid analytical errors due to partial volume effects.

**Figure 2:**
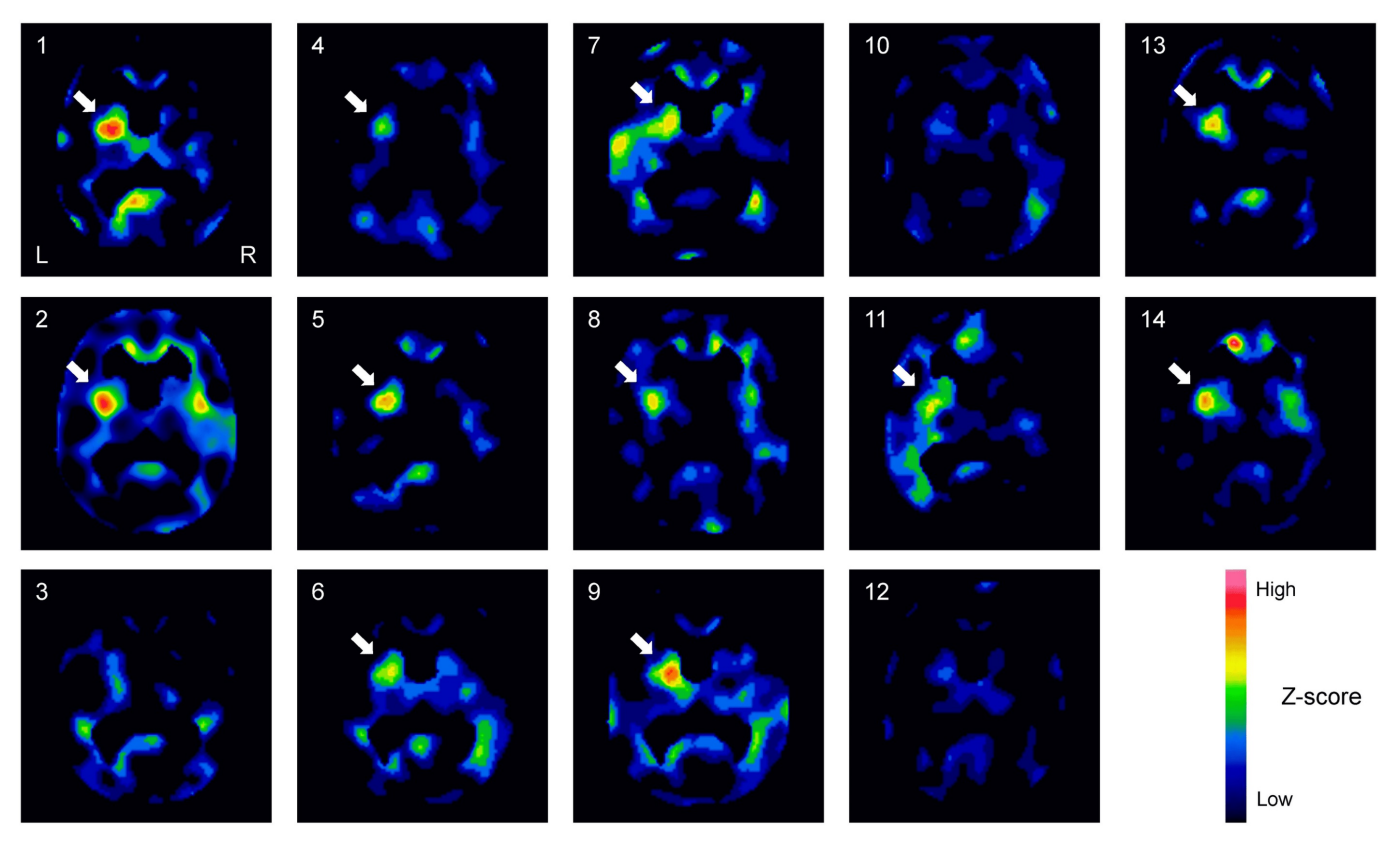
Reductions in rCBF in eZIS images. The Z-score images were normalized to the whole-brain average count (chg_snt _Z-GLOB.img) from 14 subjects in patient group 1. In the sections of the striatum and lateral ventricles, images (1, 2, 4-9, 11, 13, and 14) show decreased rCBF in the left putamen (arrows), where a higher Z-score indicates lower rCBF. rCBF: reduced regional cerebral blood flow, eZIS: easy Z-score imaging system.

In collaboration with the Department of Psychiatry at the University of Tsukuba Hospital, normal SPECT data from healthy individuals were used with written consent for the secondary use of their images. These healthy subjects were aged between 75 and 89 years (n = 48, mean age: 80.4 ± 3.8 years), which influenced the selection of age groups in the patient cohort (patient group 2). Mini-Mental State Examination (MMSE) scores [7] were obtained for all subjects. An initial observation of decreased rCBF in the left putamen using eZIS is shown in Figure 2.

Perfusion SPECT and image analysis

Cerebral perfusion was measured using 99mTc-ECD SPECT, and data were collected using a three-head gamma camera (GCA-9300R, Toshiba, Japan). For cases pertaining to the Department of Psychiatry at Tsukuba University Hospital, a multidetector SPECT machine (E. CAM; Siemens, Germany) was used to obtain the 99mTc-ECD images.

Spatial normalization and smoothing were performed using the eZIS version 4 software (Fujifilm RI Pharma Co., Ltd.; currently PDRadiopharm Inc. Tokyo, Japan). To perform interfacility comparisons, the Tottori cohort data were standardized using phantom data obtained from the University of Tsukuba Hospital. Corrections (masking and standardization) were also conducted using the eZIS software.

The processed SPECT images were analyzed using the Statistical Parametric Mapping software (SPM12, The Wellcome Center for Human Neuroimaging, UCL Queen Square Institute of Neurology, London, UK) running on MATLAB R2021a (The MathWorks, Inc., Natick, MA, USA). Statistical comparisons between patients and controls were performed on a voxel-by-voxel basis using a two-sample t-test (factorial analysis) to generate SPM (t) maps, with a height threshold of p < 0.05, corrected for multiple comparisons (family-wise error (FWE)) (Figure 2). The extent threshold was set to the smallest integer greater than or equal to the expected number of voxels per cluster. Anatomical localization was performed using the SPM anatomy toolbox (version 3.0) [8] in SPM12.

For principal component analysis (PCA), the Tottori University patient groups 1 and 2 were combined, and the Tsukuba controls were compared in the same way as described in the previous section. To obtain clearer cluster regions for analysis, the resulting maps of t-statistics were created using a height threshold of p < 0.0001 with FWE correction, and the extent threshold was set to 15 voxels. Eigenvariate values concerning decreased (dec) and increased (inc) clusters in the patient group were obtained and compared to those of the control group. The eigenvariate values represent a summary of the rCBF within a region of interest (ROI) without assuming rCBF homogeneity. These eigenvariate values were subjected to PCA. If factors (eigenvariate values) are included in the same principal component, they indicate a high correlation between them or represent common information.

Statistical analysis

Analyses were performed using IBM SPSS Statistics for Windows, Version 28.0 (IBM Corp., New York, USA). A two-tailed non-paired t-test was utilized to compare clinical and demographic characteristics between the two groups, while the *χ*^2^ test was conducted to assess sex differences between the groups. PCA was also performed using the SPSS.

## Results

Decreased rCBF area

**Figure 3:**
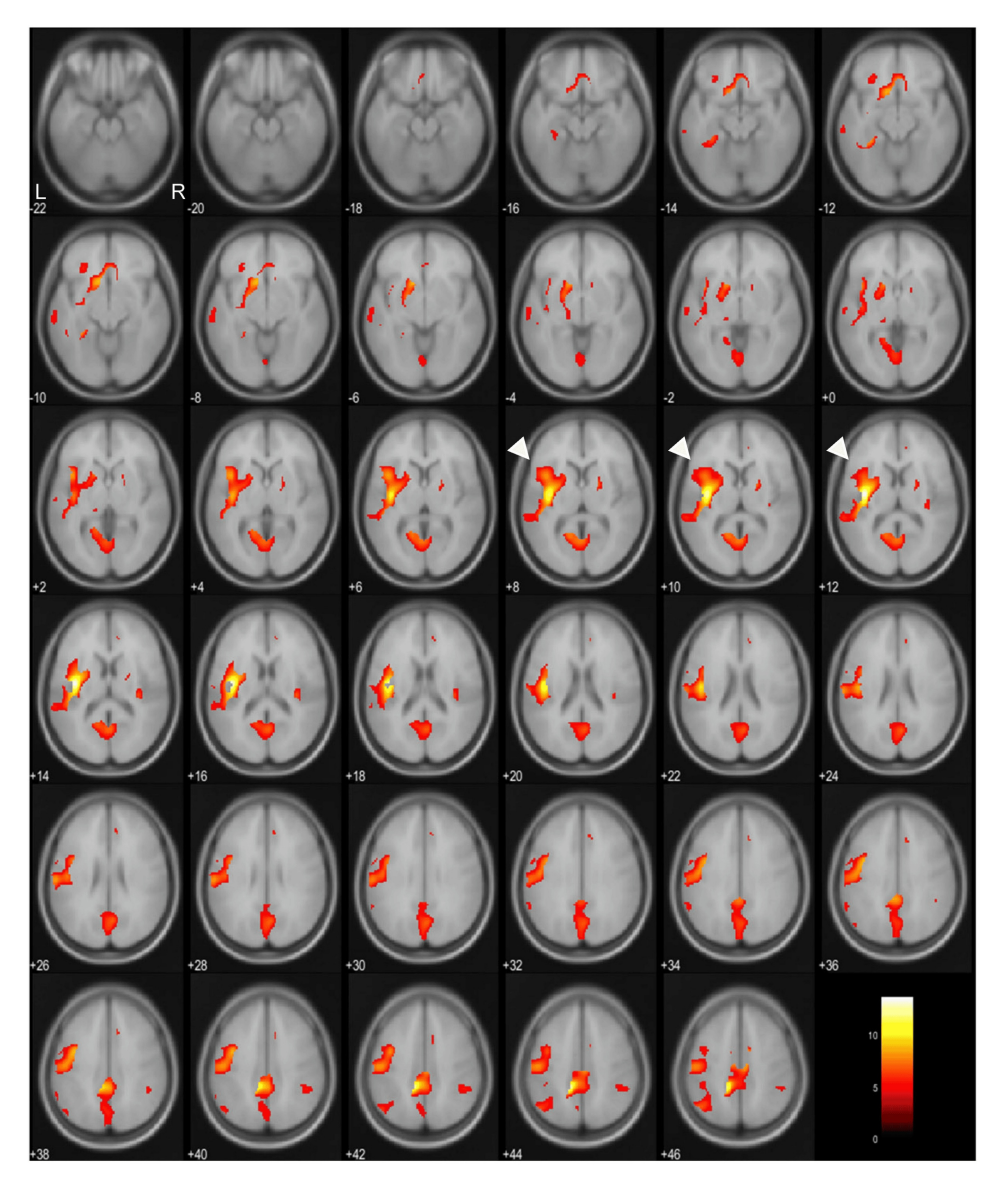
SPM analysis of decreased areas in patients compared with controls. The analysis compared patient group 2 (n = 62) with the control group (n = 48). Decreased rCBF was observed in the left insular operculum extending to the putamen (arrowheads), along with reductions in the precuneus and posterior cingulate gyrus. rCBF: regional cerebral blood flow, SPM: Statistical Parametric Mapping software.

As shown in Figure 1, we compared the rCBF between patient group 2 (Tottori cohort) and the control group (Tsukuba cohort). The sex distribution exhibited no significant differences. The SPM analysis revealed that the posterior cingulate and precuneus cortices, along with the left insular and opercular cortices and putamen, had significantly decreased rCBF (Figure 3).

Principal component

Next, we employed PCA using SPSS to investigate whether multicollinearity existed between the rCBF decreases in the precuneus/posterior cingulate and insulo-opercular-putamen areas, hypothesizing that no multicollinearity existed between them. To this end, we combined patient groups 1 and 2 (a total of 76 patients) and performed the same procedure using the same control group (Tsukuba cohort). We obtained eight decreased and seven increased areas and collected the eigenvariate values for each area (Table 1).

**Table 1:** Decreased and increased rCBF regions obtained from eigenvariate analysis. *p* FDRcorr: *p* value with false discovery rate (FDR) corrected. *p values with family-wise error rate (FWE) corrected are <0.000 in all items. dec: decreased cluster, inc: increased cluster.

Cluster No.	Coordinates (mm)			Probability for area (%)	Brain regions	Cluster size	*p* FDRcorr*	*p* FDRcorr*
	x	y	z				cluster level	peak level
dec_1	-38	-12	14	52	Insular cortex	2,683	0.000	0.000
				31	Central opercular cortex			
dec_2	-12	-40	44	39	Precuneus cortex	620	0.000	0.000
				24	Cingulated gyrus, posterior division			
dec_3	-26	-28	72	31	Postcentral gyrus	93	0.075	0.000
				25	Precentral gyrus			
dec_4	-30	-44	-10	34	Temporal occipital fusiform cortex	41	0.317	0.000
				25	Lingual gyrus			
dec_5	-14	-54	6	54	Precuneus cortex	601	0.000	0.000
				18	Cingulate gyrus, posterior division			
dec_6	20	0	10	24	Right putamen	29	0.450	0.001
dec_7	-46	-62	48	57	Lateral occipital cortex, superior division	88	0.075	0.001
				21	Angular gyrus			
dec_8	-62	-38	12	31	Superior temporal gyrus, posterior division	17	0.705	0.214
				12	Planum temporale			
inc_1	18	-88	8	14	Occipital pole	15,674	0.000	0.000
				10	Intracalcarine cortex			
inc_2	22	36	30	17	Frontal pole	1,462	0.000	0.000
				8	Superior frontal gyrus			
inc_3	-18	50	4		No match	509	0.000	0.000
inc_4	26	-22	0		No match	250	0.000	0.000
inc_5	56	-6	-20	32	Middle temporal gyrus, anterior division	116	0.001	0.000
				22	Middle temporal gyrus, posterior division			
inc_6	46	-64	40	63	Lateral occipital cortex, superior division	0.090	0.165	0.001
				5	Angular gyrus			
inc_7	56	-24	-26	48	Inferior temporal gyrus, posterior division	0.219	0.345	0.352
				6	Middle temporal gyrus, posterior division			

Notably, the left insulo-opercular and putamen (dec_1) were categorized in the first component, together with the posterior cingulate and precuneus (dec_2 and dec_5) in the PCA (Table 2). Hypoperfusion in the right putamen (dec-6) was also observed in the first component (Table 2). Therefore, we believe that these factors captured the data's main variation and correlated with each other.

**Table 2:** Component matrix using decreased and increased regions. The coefficients with high contributions to each component are shown in bold.

	Component 1	Component 2	Component 3
dec_1	0.959	-0.011	-0.091
dec_2	0.909	-0.212	-0.064
dec_4	0.887	-0.084	0.077
dec_6	0.876	0.102	-0.053
dec_8	0.875	-0.046	-0.071
dec_5	0.866	-0.313	0.027
dec_7	0.864	-0.304	-0.102
dec_3	0.813	-0.288	-0.174
inc_4	0.577	0.065	0.265
inc_3	0.567	0.460	-0.522
inc_2	0.470	0.690	-0.348
inc_5	0.445	0.625	0.453
inc_1	0.264	-0.582	0.346
inc_7	0.404	0.569	0.495
inc_6	0.438	0.045	0.472
contribution (%)	51.46	13.91	8.82

Clinical comparison

Seventy-six patients (Tottori cohort patients 1 and 2) were divided into two groups based on the high and low eigenvariate (dec-1 in Table 1) values to further assess the decreased rCBF in the left insula, operculum, and putamen (Table 3). Here, low eigenvariate values indicate low rCBF values. The male/female ratios, MCI/early AD ratios, and MMSE scores were compared between the groups. The low-versus-high eigenvariate values were 16/22 and 14/24 in males/females and 20/18 and 18/20 in MCI/early AD, respectively, and showed no significant differences (χ^2^ test). The MMSE scores were 23.2 ± 3.8 vs. 21.4 ± 3.9. Although the low rCBF group showed 1.8 points higher MMSE average score, the difference was not significant (p = 0.61, two-sided *t*-test).

**Table 3:** Comparison between low and high rCBF groups. Plus-minus values are mean ± SD. MMSE: mini-mental examination, MCI: mild cognitive impairment, AD: Alzheimer's disease, rCBF: regional cerebral blood flow.

	Low	High
Age (year)	79.3 ± 5.4	80.9 ± 5.7
Male: female	16:22	14:24
MCI: AD	20:18	18:20
MMSE	23.2 ± 3.8	21.4 ± 3.9

## Discussion

In this study, we reported decreased rCBF in the left insula, operculum, and putamen in patients who visited our hospital with forgetfulness, which, although remarkable, has received little research attention in the past. Only a few studies have reported low perfusion in the left insulo-opercular areas in patients with AD compared with controls [2,9]. Yang et al. analyzed brain morphological changes, including cortical thickness and surface area, in patients with AD and amnestic MCI, compared them with controls, and reported that both parameters were reduced in the insula and were more prominent on the left side [10]. As a unilateral reduction described above, Parkinson’s disease (PD) and other Parkinsonian syndromes may be considered neuroimage diagnoses. Although dopamine transporter-SPECT can visually reveal presynaptic nigrostriatal dysfunction [11], cortical hypoperfusion is typically observed only in the occipital lobe in PD and DLB [3]. Corticobasal degeneration (CBD) could also contribute to hypoperfusion in the insular and operculum regions [11,12]; however, the neurological examinations did not support this diagnosis. Considering the prevalence of CBD, it is unlikely that it explains the hypoperfusion in the insulo-opercular area and putamen. Generally, reduced rCBF confined to the left hemisphere is difficult to explain by a certain disease entity.

While we did not confirm the handedness of each patient or control, it is known that the left hemisphere is the dominant hemisphere in more than 93% of the general population. The affected areas on the left side are involved in various functions, including language. The insular cortex reportedly participates in emotion and language function [13] and is involved in semantic information processing [14,15], as well as episodic [16], working [17], and retrieval [18] memory. Insular hypoperfusion correlates with the severity of delusions in AD [19]. The operculum covers the insular cortex and spans the frontal, temporal, and parietal lobes. The frontal operculum is involved in thought, cognition, and planning and consists of Broca's area (Broadman areas (BAs) 44 and 45) [20]. In contrast, the parietal operculum contains the primary gustatory cortex (BA 43), while the temporal operculum contains the primary auditory cortex (Heschl gyrus, BAs 41 and 42). Note that some of these categorizations are approximations and that some BAs span gyri. In addition to regulating movements during the preparation and execution stages, the putamen contributes to various types of learning processes beyond motor learning [21]. Moreover, functional connections have been reported between the insulo-opercular-putamen areas and the cingulate cortex [22-24] and the hippocampus [25-27]. The areas of decreased rCBF identified in this study are not localized to a single region but are distributed across the insula, operculum, and putamen, each of which has several different functions. This complexity may partially explain why this observation has often been overlooked. 

Our patient cohort did not exhibit signs of speech deficits or delusions. Furthermore, patients with low perfusion in the left insula, operculum, and putamen tended to have higher, although not significantly, MMSE scores compared with those of the patients exhibiting high rCBF (Table 3). The PCA indicated that the decrease in rCBF in this region was categorized as a major component, similar to the cingulate and precuneus areas (Table 2). Consequently, we could not ascertain whether this finding had clinical significance beyond forgetfulness. Additionally, we did not determine how the natural course of this finding changes radiologically as the disease progresses. Recently, lecanemab was approved for the treatment of early AD and MCI due to AD [28]. Prior to treatment, amyloid positron emission tomography (PET) or cerebrospinal fluid (SCF) tests are necessary to confirm amyloid pathology. Whether decreased rCBF in the left insula, operculum, and putamen is indicative of MCI or early AD remains to be confirmed.

Limitations

This study has several important limitations. In this study, the diagnoses of AD and MCI were based on clinical assessment. It is desirable to confirm AD pathology in the patient group using amyloid PET or CSF biomarkers. The research was performed with different subgroups (Tottori neurology and Tsukuba psychiatry cohorts) using different SPECT imaging systems. However, it is noteworthy that this study was initiated based on positive findings derived from a single-center study. Future prospective multicenter studies are required to verify our results.

## Conclusions

This study identified decreased rCBF in the left insula, operculum, and putamen, which may serve as an early radiological finding indicative of MCI and early AD. However, the clinical significance of this hypoperfusion beyond forgetfulness, as well as its natural radiological progression, remains unclear.
